# *ACTN3* R577X Genotype and Exercise Phenotypes in Recreational Marathon Runners

**DOI:** 10.3390/genes10060413

**Published:** 2019-05-29

**Authors:** Juan Del Coso, Victor Moreno, Jorge Gutiérrez-Hellín, Gabriel Baltazar-Martins, Carlos Ruíz-Moreno, Millán Aguilar-Navarro, Beatriz Lara, Alejandro Lucía

**Affiliations:** 1 Exercise Physiology Laboratory, Camilo José Cela University, 28692 Madrid, Spain; jhellin@ucjc.edu (J.G.-H.); jgsoares@ucjc.edu (G.B.-M.); cruizm@ucjc.edu (C.R.-M.); millan.aguilar@ufv.es (M.A.-N.); blara@ucjc.edu (B.L.); 2 Sports Research Centre, Miguel Hernandez University of Elche, 03202 Alicante, Spain; vmoreno@goumh.umh.es; 3 Sport Science Department, Francisco de Vitoria University, 28223 Madrid, Spain; 4 Faculty of Sport Sciences, Research Institute i+12, Universidad Europea de Madrid, 28670 Madrid, Spain; alejandro.lucia@universidadeuropea.es; 5 Centro de Investigación Biomédica en Red de Fragilidad y Envejecimiento Saludable, 28029 Madrid, Spain

**Keywords:** α-actinin, exercise, performance, endurance, genetics, single nucleotide polymorphism

## Abstract

**Background**: Homozygosity for the X-allele in the *ACTN3* R577X (rs1815739) polymorphism results in the complete absence of α-actinin-3 in sarcomeres of fast-type muscle fibers. In elite athletes, the *ACTN3* XX genotype has been related to inferior performance in speed and power-oriented sports; however, its influence on exercise phenotypes in recreational athletes has received less attention. We sought to determine the influence of *ACTN3* genotypes on common exercise phenotypes in recreational marathon runners. **Methods**: A total of 136 marathoners (116 men and 20 women) were subjected to laboratory testing that included measurements of body composition, isometric muscle force, muscle flexibility, ankle dorsiflexion, and the energy cost of running. *ACTN3* genotyping was performed using TaqMan probes. **Results**: 37 runners (27.2%) had the RR genotype, 67 (49.3%) were RX and 32 (23.5%) were XX. There was a difference in body fat percentage between RR and XX genotype groups (15.7 ± 5.8 vs. 18.8 ± 5.5%; effect size, ES, = 0.5 ± 0.4, *p* = 0.024), whereas the distance obtained in the sit-and-reach-test was likely lower in the RX than in the XX group (15.3 ± 7.8 vs. 18.4 ± 9.9 cm; ES = 0.4 ± 0.4, *p* = 0.046). Maximal dorsiflexion during the weight-bearing lunge test was different in the RR and XX groups (54.8 ± 5.8 vs. 57.7 ± 5.1 degree; ES = 0.5 ± 0.5, *p* = 0.044). Maximal isometric force was higher in the RR than in the XX group (16.7 ± 4.7 vs. 14.7 ± 4.0 N/kg; ES = −0.5 ± 0.3, *p* = 0.038). There was no difference in the energy cost of running between genotypes (~4.8 J/kg/min for all three groups, ES ~0.2 ± 0.4). **Conclusions**: The *ACTN3* genotype might influence several exercise phenotypes in recreational marathoners. Deficiency in α-actinin-3 might be accompanied by higher body fatness, lower muscle strength and higher muscle flexibility and range of motion. Although there is not yet a scientific rationale for the use of commercial genetic tests to predict sports performance, recreational marathon runners who have performed such types of testing and have the *ACTN3* XX genotype might perhaps benefit from personalized strength training to improve their performance more than their counterparts with other *ACTN3* genotypes.

## 1. Introduction

α-Actinin-2 and α-actinin-3 are key structural proteins in the contractile apparatus of the skeletal muscle fiber, as they bind and possibly cross-link the ends of F-actin filaments at the Z-line [1]. Whereas α-actinin-2 is ubiquitously expressed in all muscle fiber types, α-actinin-3 expression is largely restricted to fast-type muscle fibers [2]. Homozygosity for the null X-allele of the R577X polymorphism in the α-actinin-3 gene, *ACTN3*, results in the complete absence of α-actinin-3 in fast-type muscle fibers [3]. Individuals with the *ACTN3* XX genotype compensate for the deficiency of α-actinin-3 through elevated expression of α-actinin-2 in fast-type muscle fibers [4], although several specific muscle phenotypes have been related to α-actinin-3 deficiency [5].

α-Actinin-3 deficiency is believed to affect the muscle’s ability to generate rapid, forceful contractions and thus might be detrimental for the production of fast and explosive movements. This notion has been verified in almost 20 case-control studies, as recently reviewed by Houweling et al., (2018), with the frequency of the XX genotype being lower in elite athletes participating in sprint and power-based sports than in the general non-athletic population. By contrast, the RR genotype, which is associated with full expression of α-actinin-3 in fast-type muscle fibers, is highly prevalent among elite athletes in sprint/power disciplines. However, the effect of the *ACTN3* XX genotype on the sports performance of recreational athletes is unexplored. The study of such a relationship might be particularly interesting given that ~20% of the world´s population is α-actinin-3 deficient [6], and because genetic testing of this polymorphism has recently become a commercially available diagnostic test [7], which can inform exercise recommendations.

In untrained populations, *ACTN3* XX individuals produce less handgrip strength and less muscle force and power than their RR counterparts [8,9,10], but this difference is lost when the same genotypes are compared in active/trained individuals [11,12,13]. Furthermore, whereas muscle fiber composition is not affected by α-actinin-3 deficiency [14]; muscle volume [8], and especially the size of fast-type muscle fibers [15], is lower in XX than in RR counterparts. Finally, a higher response to strength training has been found in RR than in XX individuals [16], coupled with a lower signaling for muscle hypertrophy in XX subjects [14]. Given this information, it might be speculated that α-actinin-3 deficiency derived from *ACTN3* XX homozygosity might also affect force and power production in recreational athletes and affect sports performance.

In addition to affecting exercise performance, *ACTN3* genotypes might also influence exercise-induced muscle damage, particularly after endurance events such as marathon running. Indeed, the X-allele has been associated with higher levels of several markers of muscle damage after exercise in amateur athletes [17,18,19]. Conversely, a higher muscle flexibility and a superior range of motion has been reported in XX individuals versus their RR referents [20,21,22], although some authors have failed to replicate this finding [23]. Although more flexible muscles are less susceptible to eccentric exercise-induced damage [24], higher muscle flexibility values do not seem to attenuate marathon-induced muscle damage in XX runners [17,18,19]. Finally, the *ACTN3* XX genotype has been related to lower body mass and lower fat-free mass [8,23], likely due to a reduction in muscle mass as a result of smaller fast-type fiber size [15,25]. However, the effect of *ACTN3* genotypes on fat mass and body composition in sedentary and clinical populations is unclear [25,26,27], and is unknown in recreational athletes. The methodological differences in assessing these exercise phenotypes, the relatively small study samples in some investigations, and the wide range of age and fitness levels under investigation make it difficult to ascertain whether the effect of *ACTN3* genotypes is of sufficient magnitude to represent a variable that affect sports performance and training in recreational athletes, as seems to be the case in elite athlete populations.

α-Actinin-3 deficiency has also been related to positive phenotypes that would explain the perpetuation of the *ACTN3* XX genotype through natural selection in human evolution. Particularly, it has been proposed that the high frequency of the X allele in some human populations could be the result of increased metabolic efficiency, possibly enhancing the capability for endurance running [28]. This theory is supported by studies in mouse models, because a shift towards a more efficient aerobic muscle metabolism has been found in *Actn3* knockout (KO) mice [6,29]. This has fueled the notion that the X allele might act as a thrifty allele [30], although this theory has little support in humans [31]. Indeed, recent case-control investigations suggest that it is unlikely that the *ACTN3* XX genotype provides an advantage in competitive endurance running performance [32,33].

The aim of the present study was to determine the influence of *ACTN3* genotypes on common exercise phenotypes in recreational marathon runners. Our main hypothesis was that, compared with their RR counterparts, *ACTN3* XX runners would present with lower values of muscle force, but higher values of running efficiency.

## 2. Materials and Methods

### 2.1. Subjects 

One hundred thirty-six healthy experienced recreational marathon runners (116 men and 20 women) volunteered to participate in this study. Participants were either recruited by email from a group of runners that had participated in previous investigations or were recruited at the time of race registration. Inclusion criteria were as follows: Age 18–65 years; being free of any history of muscle, cardiac or kidney disorders; participating in the marathon at maximal possible intensity; and having a running experience of at least 3 years, with at least three marathons completed during this period. Exclusion criteria were: taking medications during the 2 weeks prior to competing or having had a musculoskeletal injury in the month prior to the competition. The fulfillment of inclusion/exclusion criteria was verified through an ad hoc questionnaire. Age and main morphological and physical characteristics of the participants in this investigation are shown in Table 1. Before enrollment, each participant was informed about the risks and discomforts associated with the investigation and signed an informed consent document. The study was approved by the Camilo Jose Cela University Ethics Committee (ID ACTN3 approved 18/4/2018) in accordance with the latest version of the Declaration of Helsinki. Participants’ rights and confidentiality were protected during the whole experiment, and the genetic information was used only for the purposes included in this investigation.

### 2.2. Experimental Design

All participants underwent the same testing under identical experimental conditions. Participants were registered in the 2018 edition of the Rock’n’Roll Madrid Marathon and once they had completed all the testing and finished the marathon, they were included into a common database. Subsequently, participants were divided into three groups, established according to their individual *ACTN3* R577X genotype (RR, RX or XX groups). Because the men and women responded in the same manner when comparing the three genotypes, and the frequency of men/women was similar in all three groups (Table 1), we analyzed all the data without considering sex as a covariable.

### 2.3. Experimental Protocol

At least 1 week before the marathon, each participant received information about the benefits and risks of the investigation and the standardization procedures. At this time, they filled out the pre-participation ad hoc questionnaire. Participants were instructed to avoid strenuous exercise, caffeine and alcohol for the 24 h before the onset of testing, which was performed the day before the race. On this day, participants signed the informed consent and anthropometric characteristics were registered by an ISAK-certified anthropometrist following international standards [34]. Anthropometric measurements included body mass and height (±50 g scale; Radwag, Radom, Poland), skinfold thickness (±0.1 mm skinfold caliper, Holtain Ltd., Crosswell, UK: triceps, subscapular, iliac crest, abdominal, anterior and posterior thigh and medial calf) and thigh circumference (±0.5 mm fiber glass measuring tape; Holtain Ltd.: above the knee, at the maximum thigh circumference and at the gluteal furrow). Three measurements were obtained on the dominant side of the body and the mean was used for data analysis. Relative adiposity (in %) was calculated from the sum of skinfolds [35]. The mean fat-free volume of the dominant thigh (in mL/kg) was measured according to the protocol described by Jones & Pearson (1969) and normalized by body mass to allow a better comparison among groups [36].

Participants underwent a standardized 10-min warm-up including low-intensity running at 8 km/h on a treadmill. Treadmill velocity was progressively increased until 10 km/h and oxygen uptake (VO_2_) and carbon dioxide production (VCO_2_) were measured at this velocity for 5 min. Expired gases were collected breath-by-breath with a metabolic cart (Metalyzer 3B, Cortex, Leipzig, Germany), and gas exchange data of the last minute was used as a representative value. Certified calibration gases (16% O_2_, 5% CO_2_, Cortex) and a 3-L syringe were used to calibrate the gas analyzer and the flowmeter, respectively. Gas measurements were made with the clothes and shoes used during the marathon competition. The energy cost of running (in J/kg/m) was calculated using the non-protein respiratory quotient [37] and was normalized by body mass to allow a better between-subject comparison [38].

After 5 min of recovery, participants performed two maximal countermovement vertical jumps on a force platform (Quattrojump, Kistler, Wintherthur, Switzerland), as previously described [17]. The jumps were separated by a 1-min rest period. The jump with the highest height (in cm) was used for statistical analysis. Then, participants performed a whole-body isometric force test [39]. The isometric muscle strength was measured using a hand-held pull gauge (Isocontrol, Isometrico, Madrid, Spain) set at a frequency of 1000 Hz. For this measurement, participants were asked to stand on a 50 × 50 cm iron base connected to a handle-bar by a non-elastic cable. The isometric gauge was inserted within the cable, and the height of the cable was individually set to provide a 135° knee flexion while the back and the arms were completely extended. Participants were instructed to perform a maximal pull for 4 seconds and the peak value was used for analysis. The force obtained (in Newtons, N) was normalized to body mass (i.e., N/kg) to allow for a better comparison among genotypes. Thereafter, participants performed a maximal handgrip strength test with both hands (dominant and non-dominant) using a handgrip dynamometer (Grip-D, Takei, Japan). Performance was expressed in N and two attempts were performed with each hand; the peak value was used for statistical analysis.

The lunge test was performed as a measure of dorsiflexion range of motion [40]. Participants placed their foot along a measuring tape on the floor with both their big toe and heel on the centerline of the measuring tape while they leaned on a wall. The weight-bearing lunge test was performed with both limbs and the maximal dorsiflexion during the test was defined as the maximum distance of the toe from the wall while maintaining contact between the wall and knee without lifting the heel. Participants were then asked to progressively move their knee forwards while they were reclined on the wall, repeating the lunge movement until the maximum distance at which they could tolerably lunge their knee to the wall without heel lift was found [41]. At this point, dorsiflexion range of motion was performed using a handheld manual goniometer (Baseline^®^, The Therapy Connection Inc, Windham, NY, USA) by placing the center of the goniometer just below the lateral malleolus of the ankle, with one arm lined up through the lateral aspect of the fibula and the other arm lined up with the fifth metatarsophalangeal joint [42]. The measurement was repeated three times and the maximal ankle dorsiflexion (in °) was used for analysis.

On the day of the race, participants had their usual pre-competition meal at least 3 h before the race, which was not standardized among participants to avoid affecting their individual pre-competition routine. Runners were encouraged to ingest 500 mL of water 2 h before the start of the race to increase the likelihood of being euhydrated at the start line. During the race, participants wore a race bib with a time-chip to calculate the actual amount of time that it took them from the start line of the race to the finish line (net time, in min). Participants completed the race at their own pace and drank ad libitum at the hydration stations placed at 5-km intervals with no indications about running pace or fluid and food strategies. The marathon race was held in April on a sunny day with a mean dry temperature of 21.0 ± 2.1 °C (range 15–26 °C, temperature readings at 30-min intervals from 0- to 5-h after the race onset) and a mean relative humidity of 43 ± 2% (range 40–51%).

### 2.4. Genetic Testing

Genomic DNA was isolated using an organic-based DNA extraction method adapted to Amicon^®^ (Sigma-Aldrich, Madrid, Spain) Ultra 0.5-mL columns, including a final concentration step to 50 µL [43]. To avoid contamination, recommendations for molecular genetics laboratories were followed, including physically-isolated work area laboratories for each process (sample manipulation and extraction). In addition, reference samples (internal controls, blank samples and negative controls) and contamination monitoring in all steps were included. Positive controls for all genotypes were obtained from the Mexican branch of the CANDELA Consortium [44]. Genotyping of *ACTN3* rs1815739 polymorphism (c.1858C>T; p.R577X) was conducted using a TaqMan SNP Genotyping Assay (Assay ID: C___590093_1_; Applied Biosystems, Foster City, CA, USA) and the reaction was performed in an Applied Biosystems 7500 Fast Real-Time PCR System (Applied Biosystems). The results were analyzed using 7500 Software v2.0.5 (Applied Biosystems).

### 2.5. Statistical analysis

The difference in the distribution of men/women in each genotype group was tested with crosstab and Chi square tests, including adjusted standardized residuals. The normality of the remaining variables was initially tested with the Shapiro-Wilk test and all variables showed a normal distribution. Group comparisons (RR vs. RX vs. XX) were performed using one-way analysis of variance (ANOVA). When the ANOVA showed a significant group-effect, between-group differences were assessed using the Tukey post-hoc test. The significance level was set at 0.05. The effect size (ES) for each full ANOVA analysis was calculated using the Eta squared (η^2^) by using between-groups sum of squares and the total sums of squares for all ES. The magnitude of η^2^ was interpreted following the guidelines by Cohen [45] as follows: small: 0.01; medium = 0.06; large: 0.14. Data are presented as mean ± standard deviation (SD) and all the analyses were performed with the statistical package SPSS version 20.0 (SPSS Inc., Chicago, IL, USA). The ES was also calculated in all pairwise comparisons, by using the Hedges’ *g* ± 95% confidence intervals (CI), to assess the magnitude of the between-group differences in the phenotypes under investigation. ES were interpreted according to the following ranges: <0.2, trivial; 0.2–0.6, small; 0.6–1.2, moderate; 1.2–2.0, large; 2.0–4.0, very large; and >4.0, extremely large [46].

## 3. Results

The genotyping success rate was 99%. From the study sample of 136 runners, 27.2% were genotyped as *ACTN3* RR, 49.3% were RX and 23.5% were XX. Participants had similar running experience, best race time in marathon, number of completed marathons in the three previous years, and comparable training characteristics (Table 1). In addition, the net race time in the investigated marathon was similar for all three genotypes (236 ± 36, 236 ± 44, 244 ± 27 min, respectively; *p* = 0.509, η^2^ = 0.01).

**Figure 1:**
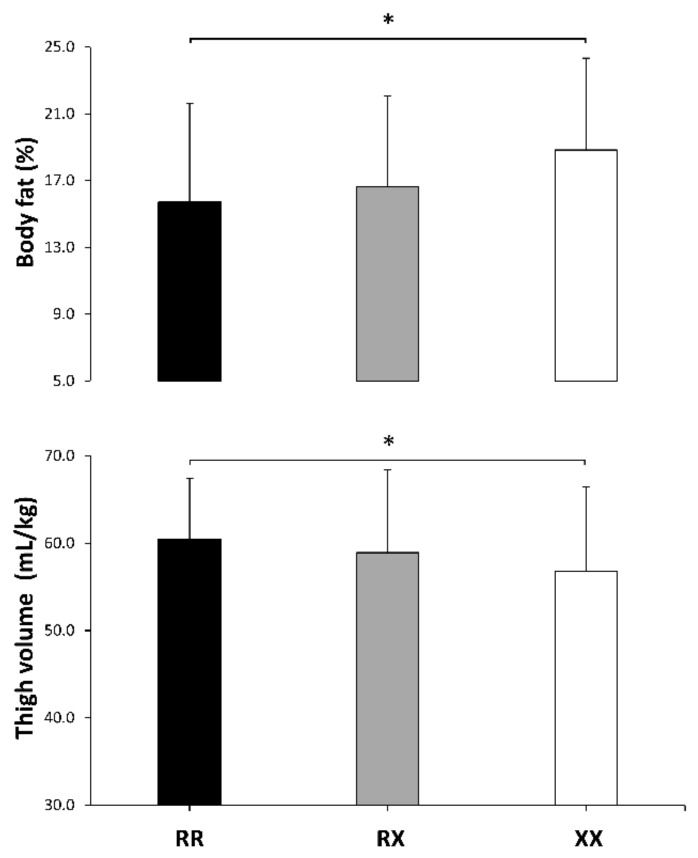
Body fat percentage and thigh fat-free volume in *ACTN3* RR, RX and XX recreational marathon runners. Data are mean ± standard deviation (SD) for each genotype. (*) Differences identified by a post-hoc analysis at *p* < 0.05.

**Figure 2:**
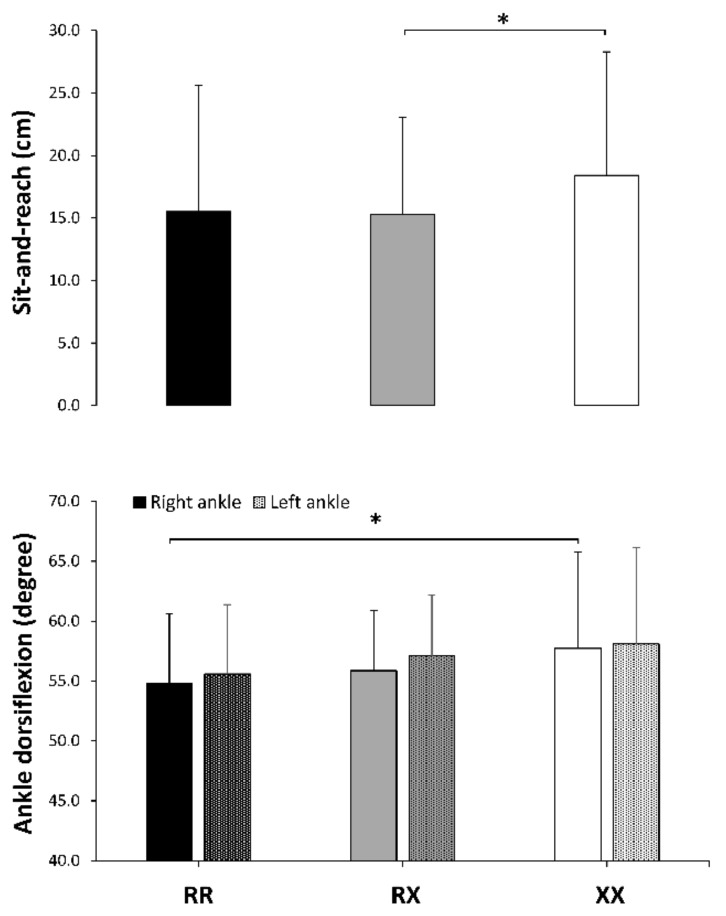
Distance reached in the sit-and-reach test and maximal ankle dorsiflexion in the lunge test in *ACTN3* RR, RX and XX recreational marathon runners. Data are mean ± SD for each genotype. (*) Differences identified by a post-hoc analysis at *p* < 0.05.

The ANOVA post-hoc analyses revealed that body fat percentage was higher in the XX than in the RR group (Figure 1, upper panel), although the differences between XX and the RR or RX group were small–moderate (Table 2). The ANOVA post-hoc analyses only revealed a small-to-moderate difference between RR and XX groups for thigh volume in the dominant leg (Figure 1, lower panel). By contrast, performance in the sit-and-reach-test was higher in the XX group than in the RX group (Figure 2, upper panel). Post-hoc analyses only revealed an RR vs. XX difference in the right ankle for maximal dorsiflexion during the weight-bearing lunge test (Figure 2, lower panel) with no differences between groups in the left ankle (Table 2).

**Figure 3:**
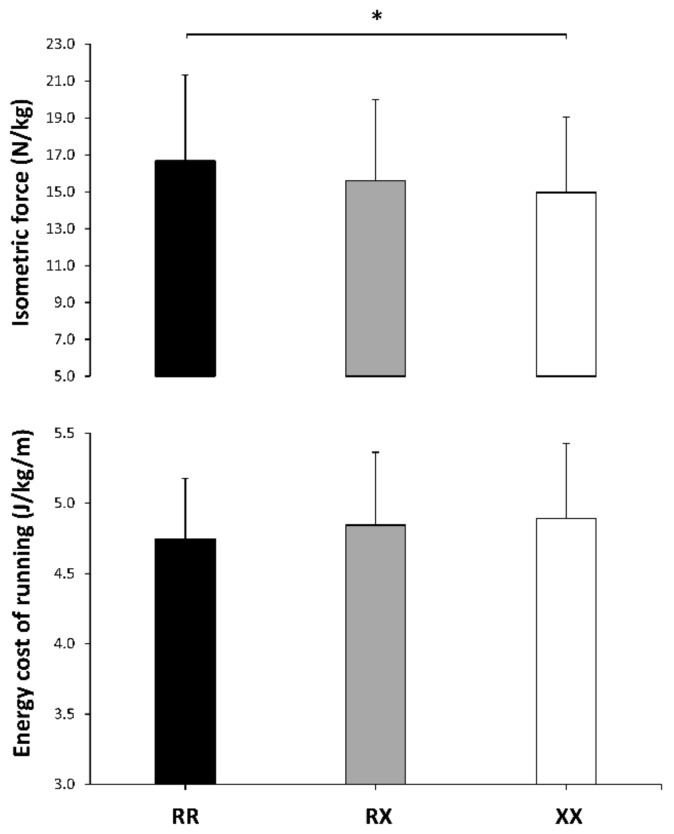
Maximal isometric force and energy cost of running in *ACTN3* RR, RX and XX recreational marathon runners. Data are mean ± SD for each genotype. (*) Differences identified by a post-hoc analysis at *p* < 0.05.

There were no differences in handgrip force between the genotypes (Table 3). However, the RR group had a higher isometric force relative to body mass than the XX group (Figure 3, upper panel). Jump height during a countermovement jump was similar between the genotype groups (Table 3). During the running test at 10 km/h on a treadmill, the post-hoc analyses did not reveal any difference in tidal volume or respiratory rate, although the differences in respiratory rate between XX and the other two genotype groups were small–moderate. As a result, pulmonary ventilation at 10 km/h was lower in the RR than in the XX group with a difference of moderate magnitude (Table 3). Post-hoc analyses did not reveal any between-groups difference for VO_2_, respiratory exchange ratio or for the energy cost of running (Figure 3, lower panel and Table 3).

## 4. Discussion

**Figure 4:**
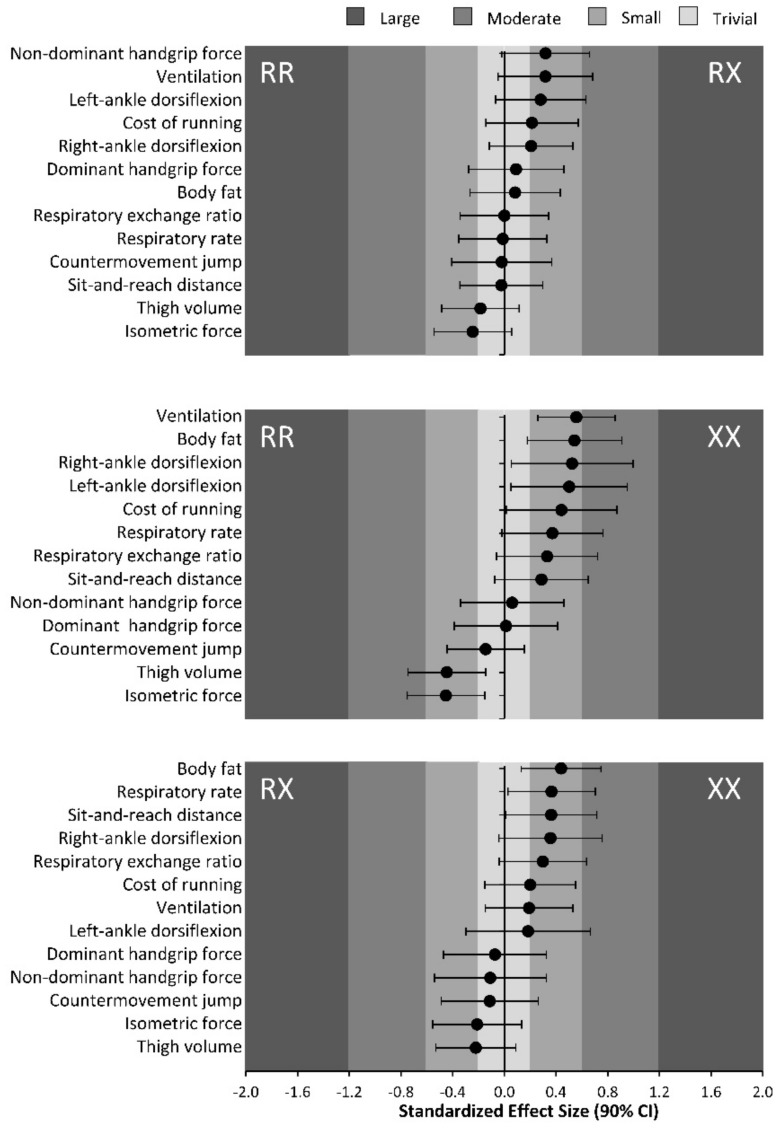
Magnitude of the *ACTN3* genotype effect, calculated with Hedges’ *g* ± 95% confidence interval, on the phenotypes investigated for the comparison of RR vs. RX (upper panel), RR vs. XX (middle panel), and RX vs. XX (lower panel) genotypes. Negative values indicate higher values of the variable for the group categorized at the left. Positive values indicate higher values of the variable for the group categorized at the right.

The *ACTN3* R577X genotype is a well-characterized polymorphism that can affect physical and sports performance in elite athletes. Homozygosity for the X allele has been deemed a deleterious trait for success in elite sprint- and power-based sports, whereas the RR genotype has been considered propitious for the production of forceful and speedy contractions and thus optimal elite sport [1]. This information is being directly applied to recreational or less well-trained athletes without the knowledge of whether this polymorphism, and the consequent reduction/absence of α-actinin-3, affects muscle function in these populations as it does elite performers. Accordingly, our aim was to determine the influence of the three *ACTN3* genotypes on common exercise phenotypes in recreational marathon runners. As main outcomes, we found that the genotype frequencies of the *ACTN3* R577X polymorphism in our sample of marathoners was very similar to the ones previously reported in Spanish sedentary controls [47]. However, XX runners likely had higher body fat percentage, muscle flexibility and ankle dorsiflexion than RR or RX runners, despite similar age, running experience and training characteristics. Moreover, XX runners likely presented lower whole-body muscle force production and lower thigh fat-free mass volume than their RR counterparts. On the other hand, the *ACTN3* genotype did not affect the energy cost of running or the time necessary to complete a marathon. Overall, these data indicate that the R577X polymorphism might affect several characteristics in recreational endurance runners. Specifically, the outcomes of this investigation suggest that α-actinin-3-deficient runners might have lower strength values, but this does not translate into a poorer marathon performance. Although the magnitude of the *ACTN3* genotype effect on the exercise phenotypes investigated here was small-to-moderate in most cases, the consistency of the differences between the XX runners and the other two genotypes suggests a likely effect of α-actinin-3 deficiency on force production, muscle mass and muscle flexibility in endurance runners (Figure 4).

A myriad of case-control studies has confirmed that homozygosity for the X allele hinders success in elite speed sports [2]. Nevertheless, these types of investigations do not allow for causal conclusions or explanations for this genetic influence on performance. To better understand the effect of α-actinin-3 deficiency, MacArthur et al. [6] generated an *Actn3* knock-out (KO) mouse, which recapitulates the features of human α-actinin-3 deficiency through loss of the expression of this protein in fast-type skeletal muscle fibers, while at the same time showing elevated expression of α-actinin-2. *Actn3*-KO mice have lower grip strength [29] and inferior fast force muscle production [48] than their wild-type littermates, which might explain in part the low frequency of XX in the pool of elite athletes in speed sports. In addition, *Actn3*-KO mice have less body weight due to lower lean body mass [25], with no changes in fat mass.

Interestingly, some of the main phenotypes found in the *Actn3*-KO mouse model have also been found in our sample of recreational runners, as XX runners likely presented lower values of maximal isometric force, lower fat-free volume and higher body fat percentage than RR or RX runners (in this case, without any effect on body mass). While the lack of α-actinin-3 has been related to these phenotypes in untrained individuals [8,9,49], this is the first study to show these negative effects on a sample of trained and competitive individuals, which might mirror the situation in elite athletes.

A summary of how the *ACTN3* genotype alters muscle function during exercise has been recently performed by Lee et al. [50] using the main outcomes found in the *Actn3*-KO mouse model. Briefly, α-actinin-3 interacts with a number of partner proteins, which broadly fall into three biological pathways: structural, metabolic and signaling. First, α-actinin-3 is a structural protein with a role for attaching and cross-linking actin filaments, and thus, its deficiency might negatively affect the structure of the sarcomere as well as its ability to produce force during muscle shortening [29]. As an additional cause for the lower values of force associated with the XX genotype, it has also been found that *Actn3* KO mice have a lower lactate dehydrogenase [6] and glycogen phosphorylase enzyme activity [51] in fast twitching muscle fibres. These enzymatic changes are consistent with a lower ability to catabolize glycogen into glucose and a subsequent reduction in the capacity to convert glucose into lactate in skeletal muscle fibres, which would be a limiting metabolic factor for high-intensity/fast muscle contractions. The spectrum of changes within muscle fibers associated with α-actinin-3 deficiency also includes a higher calcineurin activity, which is found in both *Actn3*-KO mice and humans with the XX genotype [52]. In this context, the muscle tissue of α-actinin-3-deficient individuals might be theoretically more prone to adapt to endurance training stimuli rather than to strength- or power-oriented programs [53]. Thus, based on the present findings, and those of the animal model, it might be assumed that the *ACTN3* XX genotype is likely associated with lower muscle strength. Whether these effects can be offset with individualized training warrants further investigation. In addition, exploring the relationship between α-actinin-3 deficiency and body fatness requires further analysis in active, untrained and obese populations, because, to date, this link is unclear [25].

It has been hypothesized that the X allele might have helped in the adaptation to environments with scarce food resources, where a more efficient muscle metabolism and lower cost of locomotion would be essential for survival [54]. Indeed, this theory was confirmed in the *Actn3*-KO mouse model, which showed a shift towards more efficient and aerobic muscle metabolism [6,29]. In humans, higher VO_2peak_ and higher running speed at the ventilatory threshold have been reported in XX versus RR counterparts [55,56]. However, a study designed to relate the XX genotype to a lower cost of locomotion found that the RX genotype was more efficient for running [31]. The present investigation also disputes this association, because the energy cost of running at 10 km/h was similar in all three genotypes groups. The lack of a lower cost of running in XX runners compared with R-allele carriers, might be related to another phenotype present in runners with α-actinin-3 deficiency, that is, a higher muscle flexibility and ankle range of motion (Table 2). Indeed, the less flexible runners might also be the most economical when running [57,58], suggesting that low muscle flexibility in certain areas of the musculoskeletal system may enhance running economy in sub-elite male runners. Thus, it is possible that the higher muscle flexibility of XX runners offsets the metabolic effect of α-actinin-3 deficiency, although this suggestion requires further investigation. In addition, XX runners needed greater pulmonary ventilation values than RR counterparts to meet the metabolic demands of running at 10 km/h. This finding is a novelty of this investigation as no previous investigation has reported changes in pulmonary ventilation across the three different *ACTN3* genotypes. Although the cause for a higher ventilation in XX is not evident from our data, this might be the result of a lower running speed at the respiratory compensation threshold. In any case, the explanation of an effect of the *ACTN3* genotype on the mechanics of ventilation during running, if any, merits further research. Overall, our findings suggest that the 577X allele does not increase running economy and, thus, the persistence of this null allele might be explained beyond its potential metabolically thrifty properties, as previously suggested [26].

An alternative hypothesis for the survival of the 577XX genotype emerges from our data and previous research. Higher values of muscle flexibility and range of motion have been found for XX individuals in different joints [20,21,22], which, although negative for running economy, might confer enhanced muscle function. In the present study, likely higher trunk flexibility and ankle dorsiflexion values were identified in XX runners, which might be associated with a higher ground-force application [59] and lower leg stiffness during running [60]. In addition, higher muscle flexibility might produce a protective role against muscle damage during exercise [24]. However, despite the potential benefits of the enhanced muscle flexibility exhibited by our XX runners, previous evidence suggests that running economy or reduction of exercise-induced muscle damage are not improved by α-actinin-3 deficiency [1]. Lastly, it has been suggested that the *ACTN3* genotype affects α-actinin-3 expression in a dose-dependent fashion, indicating that RX individuals might have intermediate phenotypes [61]. Interestingly, this notion is reinforced by the present data because RX individuals were at the mid-point for isometric muscle strength, body fatness, thigh volume, muscle flexibility, and ankle dorsiflexion (Figure 1, Figure 2 and Figure 3), while the magnitude for the effect with respect to XX runners was essentially lower than when comparing RR and XX genotypes (Figure 4). Thus, both the XX and RX genotype can be related to some favorable phenotypes for endurance running, although their utility should be consolidated with further research.

The present investigation does have some limitations. First, we recruited a convenience sample of 136 marathoners that were registered in a competitive marathon. After grouping by genotype, we found that groups were similar in the distribution of sexes, age, running experience, and training characteristics. However, there was high intersubject variability even within each group. Thus, further investigation with larger and more homogeneous samples and controlled training habits should be performed to confirm these outcomes. Also, we used a battery of testing to identify the effect of *ACTN3* genotypes on several exercise phenotypes of utility for endurance runners; however, other phenotypes such as VO_2max_ and running velocity at lactate threshold should be investigated because of their high association with endurance performance [62]. Several of the major outcomes of the present investigation could have been influenced by some variables that we did not assess, such as diet and previous resistance or muscle flexibility training background. The lack of control for these potential confounders therefore represents a limitation of our investigation. Finally, the present study was focused on the influence of only one polymorphism on these phenotypes, and several other candidate genes might have exerted an influence on the phenotypes investigated. Despite these limitations, we believe that the investigation contributes to the current knowledge on the effect α-actinin-3 deficiency has on muscle function, exercise traits.

## 5. Conclusions

In summary, compared with their RR peers, *ACTN3* XX marathon runners likely had lower values of whole-body isometric muscle force and lower fat-free mass volume in the thigh, and a higher percentage of body fatness. By contrast, *ACTN3* XX marathon runners had higher muscle flexibility and ankle range of motion, whereas no clear genotype-effect was found for running economy, handgrip force, and jump height. Thus, α-actinin-3 deficiency is associated with several physiological and anthropometric traits in recreational endurance runners. Nevertheless, the magnitude of differences among *ACTN3* genotypes was small-to-moderate and did not affect marathon performance. Future investigations should determine whether personalized endurance training based on genetics is effective to reduce the partially negative effects of α-actinin-3 deficiency in trained athletes. Although there is not yet a scientific rationale for the use of commercial genetic tests to predict sports performance [63,64], the outcomes of this investigation suggest that those recreational marathon runners with the *ACTN3* XX genotype might perhaps benefit from personalized strength training (to compensate for their lower muscle force capacity) more than their counterparts who are carriers of the R-allele.

## Figures and Tables

**Table 1:** Age, anthropometric characteristics, running experience, and training status of marathoners with different *ACTN3* R577X genotypes. Data are mean ± standard deviation (SD) for each genotype. Degrees of freedom = 2, between-groups; 133, intra-groups; 135, total.

Variable (Units)	RR	RX	XX	*p*	η^2^
*n* (frequency)	37 (27.2%)	67 (49.3%)	32 (23.5%)	-	-
Men/women (frequency)	31/6 (83.8/16.2%)	58/9 (86.6/13.4%)	27/5 (84.4/15.6%)	0.922	-
Age (years)	41.2 ± 10.2	40.3 ± 8.8	40.7 ± 9.8	0.880	<0.01
Body mass (kg)	70.9 ± 7.1	71.6 ± 10.8	72.8 ± 10.5	0.731	<0.01
Body height (m)	1.73 ± 0.06	1.73 ± 0.08	1.72 ± 0.10	0.723	<0.01
Body mass index (kg/m^2^)	23.7 ± 1.7	23.8 ± 1.2	24.5 ± 1.5	0.26.9	0.03
Running experience (years)	9.0 ± 7.5	8.1 ± 7.8	8.3 ± 6.0	0.880	<0.01
Best race time in the marathon (min)	218 ± 27	223 ± 38	219 ± 39	0.881	<0.01
Completed marathons (number)	5 ± 4	5 ± 3	5 ± 3	0.776	<0.01
Average training distance /week (km)	50.7 ± 14.6	52.5 ± 17.0	51.7 ± 16.9	0.889	<0.01
Training sessions /week (number)	4 ± 1	4 ± 1	4 ± 1	0.794	<0.01

**Table 2:** Body fatness, thigh volume, distance obtained in the sit-and-reach test, and ankle dorsiflexion in *ACTN3* RR, RX and XX recreational marathon runners. Data are mean ± SD for each genotype. ES = Effect size calculated with Hedges’ *g*; CI = Confidence interval. Degrees of freedom = 2, between-groups; 133, intra-group; 135, total.

Variable (Units)	RR	RX	XX	ES ± 95% CIRR vs. RX	ES ± 95% CIRR vs. XX	ES ± 95% CIRX vs. XX	*p*	η^2^
Body fat (%)	15.7 ± 5.8	16.2 ± 6.1	18.8 ± 5.5	0.1 ± 0.3	0.5 ± 0.4	0.4 ± 0.3	0.024	0.06
Thigh volume (mL/kg)	60.5 ± 6.9	58.9 ± 9.5	56.8 ± 9.6	−0.2 ± 0.3	−0.4 ± 0.3	−0.2 ± 0.3	0.043	0.05
Sit-and-reach test (cm)	15.5 ± 10.1	15.3 ± 7.8	18.4 ± 9.9	0.0 ± 0.3	0.3 ± 0.4	0.4 ± 0.4	0.046	0.04
Right-ankle dorsiflexion (degrees)	54.8 ± 5.8	55.9 ± 5.0	57.7 ± 5.1	0.2 ± 0.3	0.5 ± 0.5	0.4 ± 0.4	0.044	0.05
Left-ankle dorsiflexion (degrees)	55.6 ± 4.8	57.1 ± 5.6	58.1 ± 5.1	0.3 ± 0.3	0.5 ± 0.5	0.2 ± 0.5	0.103	0.03

**Table 3:** Handgrip force, isometric force, countermovement jump height, and respiratory exchange data while running at 10 km/m, in *ACTN3* RR, RX, and XX marathon runners. Data are mean ± SD for each group. ES = Effect size calculated with Hedges’ *g*; CI = Confidence interval; VO_2_ = oxygen uptake. Degrees of freedom = 2, between-groups; 133, intra-group; 135, total.

Variable (Units)	RR	RX	XX	ES ± 95% CIRR vs. RX	ES ± 95% CIRR vs. XX	ES ± 95% CIRX vs. XX	*p*	η^2^
Non-dominant handgrip force (N)	388 ± 73	402 ± 79	393 ± 91	0.3 ± 0.3	0.1 ± 0.4	−0.1 ± 0.4	0.663	<0.01
Dominant handgrip force (N)	414 ± 67	421 ± 81	415 ± 89	0.1 ± 0.4	0.0 ± 0.4	−0.1 ± 0.4	0.897	<0.01
Isometric force (N/kg)	16.7 ± 4.7	15.6 ± 4.4	14.7 ± 4.0	−0.2 ± 0.3	−0.5 ± 0.3	−0.2 ± 0.3	0.038	0.03
Countermovement jump height (cm)	26.9 ± 4.2	26.8 ± 5.3	26.2 ± 5.4	0.0 ± 0.4	−0.1 ± 0.3	−0.1 ± 0.4	0.818	<0.01
Tidal volume (L/respiration)	2.3 ± 0.7	2.5 ± 0.6	2.3 ± 0.6	−0.2 ± 0.3	0.0 ± 0.4	−0.2 ± 0.3	0.388	0.01
Respiratory rate (respirations/min)	28.7 ± 7.7	28.6 ± 8.7	31.8 ± 8.9	0.0 ± 0.3	0.4 ± 0.4	0.4 ± 0.3	0.188	0.03
Ventilation (L/min)	62.0 ± 9.5	65.8 ± 13.1	68.3 ± 12.9	0.3 ± 0.4	0.6 ± 0.3	0.2 ± 0.3	0.012	0.09
VO_2_ (mL/kg/min)	38.4 ± 3.3	39.0 ± 3.9	39.3 ± 4.1	0.2 ± 0.4	0.2 ± 0.4	0.1 ± 0.4	0.389	<0.01
Respiratory exchange ratio	0.89 ± 0.06	0.89 ± 0.07	0.91 ± 0.06	0.0 ± 0.3	0.3 ± 0.4	0.3 ± 0.3	0.130	0.02
Energy cost of running (J/kg/m)	4.7 ± 0.4	4.8 ± 0.5	4.9 ± 0.5	0.2 ± 0.4	0.4 ± 0.4	0.2 ± 0.4	0.448	0.01
